# Human *Lactobacillus* Biosurfactants as Natural Excipients for Nasal Drug Delivery of Hydrocortisone

**DOI:** 10.3390/pharmaceutics14030524

**Published:** 2022-02-26

**Authors:** Elisa Corazza, Angela Abruzzo, Barbara Giordani, Teresa Cerchiara, Federica Bigucci, Beatrice Vitali, Massimiliano Pio di Cagno, Barbara Luppi

**Affiliations:** 1 Department of Pharmacy and Biotechnology, Alma Mater Studiorum, University of Bologna, Via San Donato 19/2, 40127 Bologna, Italy; elisa.corazza7@unibo.it (E.C.); barbara.giordani4@unibo.it (B.G.); teresa.cerchiara2@unibo.it (T.C.); federica.bigucci@unibo.it (F.B.); b.vitali@unibo.it (B.V.); barbara.luppi@unibo.it (B.L.); 2 Department of Pharmacy, Faculty of Mathematics and Natural Sciences, University of Oslo, Sem Sælands vei 3, 0371 Oslo, Norway; m.p.d.cagno@farmasi.uio.no

**Keywords:** biosurfactants, *Lactobacillus*, nasal delivery, drug solubility, mucoadhesion, drug permeation, mucin layer

## Abstract

The inclusion of a chemical permeation enhancer in a dosage form is considered an effective approach to improve absorption across the nasal mucosa. Herein we evaluated the possibility of exploiting biosurfactants (BS) produced by *Lactobacillus gasseri* BC9 as innovative natural excipients to improve nasal delivery of hydrocortisone (HC). BC9-BS ability to improve HC solubility and the BS mucoadhesive potential were investigated using the surfactant at a concentration below and above the critical micelle concentration (CMC). In vitro diffusion studies through the biomimetic membrane PermeaPad^®^ and the same synthetic barrier functionalized with a mucin layer were assessed to determine BC9-BS absorption enhancing properties in the absence and presence of the mucus layer. Lastly, the diffusion study was performed across the sheep nasal mucosa using BC9-BS at a concentration below the CMC. Results showed that BC9-BS was able to interact with the main component of the nasal mucosa, and that it allowed for a greater solubilization and also permeation of the drug when it was employed at a low concentration. Overall, it seems that BC9-BS could be a promising alternative to chemical surfactants in the nasal drug delivery field.

## 1. Introduction

The nasal route has been conventionally used for delivery of drugs aimed to treat local diseases, but it was also demonstrated to be potentially exploited as an alternative way of systemic administration [1,2]. Underlying the growing consideration towards nasal delivery are the advantages that it offers over conventional systemic delivery strategies [3,4]. Among these, it is worth mentioning its non-invasive character, its easy accessibility for the administration of drugs and a comparatively high drug uptake in the systemic circulation thanks to the great vascularization of the respiratory nasal mucosa. Furthermore, intranasal administered systemically acting drugs are subjected to a minor presystemic metabolism with a consequent higher bioavailability, and for those drugs intended to target the brain there is the possibility to directly access the cerebrospinal fluid by-passing the blood–brain barrier [3,4,5].

Despite the numerous advantages of nasal delivery, there are also shortcomings to be considered, such as the restricted nasal tissue surface area limiting the applicable volume of formulations, the rapid drug clearance because of the mucociliary system, which is responsible for the low drug retention time in the nasal cavity, and also the enzymatic degradation [3,4,5]. Moreover, drugs delivered nasally should first overcome the physical barriers represented by the mucus gel layer and the nasal epithelium before reaching their target, either if it is local or systemic [4,5,6]. The mucus is a hydrophilic layer in which mineral salts, proteins, glycoproteins, and lipids are found. Among glycoproteins, mucins prevail, thus contributing to the mucus layer viscosity, to its negative charge and its mesh-like structure [7,8]. As a result, the mucus layer acts as a barrier towards the diffusion of foreign entities, drugs included. The absorption of active pharmaceutical ingredients is also limited by the nasal epithelium which, being composed of pseudostratified columnar cells interconnected via tight junctions, acts as a second barrier. Based on their chemical features, drugs can overcome the epithelium through different strategies, such as transcellular diffusion, or partitioning across the membrane via a concentration gradient, that is characteristic of small hydrophobic molecules, but also activating some sorts of active transport that are usually required for the absorption of hydrophilic drugs [6].

Because of the difficulties involved in nasal delivery, auxiliary agents are needed to overcome these limits [4]. Although many approaches have been investigated to improve permeation of drugs through the nasal mucosa, the most frequently employed is the inclusion of absorption enhancers in the formulation, such as surfactants [5,6]. The latter are amphiphilic molecules able to enhance drug absorption in different ways: perturbing the cell membrane, transiently opening the tight junctions, or preventing the enzymatic degradation of drugs [6,9]. Among the different molecules that belong to the group of permeation enhancers classified as surfactants are biosurfactants (BS) [6]. BS are drawing interest because they better suit the current trend of the scientific community that is looking for more eco-friendly materials obtainable from natural resources [9,10,11]. In fact, microorganisms like yeasts, bacteria and some filamentous fungi can produce different substances, BS included, just using a set of carbon sources and energy for growth [9]. Along with their being natural compounds, what makes BS very appealing from an industrial point of view, are some features that make them more advantageous compared to chemical and synthetic surfactants. Indeed, BS are biodegradable molecules with a good safety profile and great surface, interfacial and emulsifying activity. BS also show an excellent tolerance towards temperature, pH and ionic strength; furthermore, they exert a broad spectrum of biological activities useful for biomedical and pharmaceutical applications [9,10,11,12,13].

Abruzzo et al. have recently isolated a novel biosurfactant from the human strain *Lactobacillus gasseri* BC9 [14], that is a probiotic bacterium with a positive influence on human health [15,16] and which does not require a biosafe environment for its handling. BC9-BS consists of peptide-like molecule, whose hydrophobic moiety is made of hydrocarbon chains of different length, whereas the hydrophilic moiety comprises the aminoacidic residues His, Val and Thr [14]. This lipopeptidic biosurfactant exhibits surface active properties with a critical micelle concentration (CMC) around 2 mg/mL and good emulsification activity [14]. Moreover, BC9-BS in vitro cytotoxicity has also been studied on both human and murine fibroblasts demonstrating that it does not affect cell viability when used at concentrations up to five-fold its CMC [17]. Considering the interesting properties of this natural surfactant, some of its possible applications in the pharmaceutical field have already been investigated. BC9-BS was demonstrated to be potentially exploited as therapeutic agent to counteract infections caused by methicillin resistant *Staphylococcus aureus* biofilms [17], and it was also employed as green excipient in drug formulations. In fact, BC9-BS was used on the one hand to develop mixed vesicles active against chronic vaginal infections [14] and, on the other hand, as a permeation enhancer in transdermal drug delivery [18].

Based on these assumptions, we purposed to evaluate BC9-BS as an innovative natural excipient to improve nasal administration of the Biopharmaceutical Classification System class II drug hydrocortisone (HC). In vitro diffusion studies through different membranes, the biomimetic membrane PermeaPad^®^, the same barrier functionalized with a mucin layer and the sheep nasal mucosa, were performed in the presence of BC9-BS at concentrations below and above its CMC, and HC solubility was measured. Additionally, mucoadhesive studies were conducted to investigate BS interaction with mucin. The ability of BC9-BS to act as a solubilizing agent and permeation enhancer, together with its capacity to interact with the main component of the mucus layer, were then compared to those of two other surfactants: d-α-tocopheryl polyethylene glycol 1000 succinate (TPGS) as an example of the non-ionic surfactants, that together represent the most clinically advanced permeation enhancers in nasal delivery [19], and cocamidopropyl betaine (CAPB), a mild zwitterionic surfactant frequently used in cosmetic industries [20].

## 2. Materials and Methods

### 2.1. Materials

Cocamidopropyl betaine (CAPB) Amphotensid B4/C was provided from Farmalabor srl (Canosa di Puglia, Italy), whereas D-α-tocopheryl polyethylene glycol 1000 succinate (TPGS) was a kind gift from BASF SE (Ludwigshafen, Germany). Hydrocortisone, mucin type II from porcine stomach, all chemicals, and solvents were of analytical grade and purchased from Sigma-Aldrich (Milan, Italy), except for sodium chloride (NaCl) that was supplied by Carlo Erba (Milan, Italy). Phosphate buffer solution (PBS) at pH 7.4 was composed of 7.4 mM Na_2_HPO_4_·12H_2_O, 1.1 mM KH_2_PO_4_, and 136 mM NaCl. PBS at pH 5.5 was employed to simulate the pH of the nasal cavity and it was composed of 4.2 mM Na_2_HPO_4_*12H_2_O, 100 mM KH_2_PO_4_, 45.5 mM NaCl. Man, Rogosa and Sharpe (MRS) culture media and GasPak EZ were supplied by Difco (Detroit, MI, USA) and Becton Dickinson & Co. (Sparks, MD, USA), respectively. L-cysteine hydrochloride monohydrate was purchased from Merck (Darmstadt, Germany).

### 2.2. L. gasseri BC9 Cultivation and BC9 Biosurfactant Isolation

The biosurfactant (BS) produced by *Lactobacillus gasseri* BC9 was obtained following a well-established procedure designed to isolate the fraction of biosurfactant that is bound to the bacterial cell surface [17]. Briefly, 100 mL of an overnight lactobacilli culture was inoculated in 900 mL of MRS broth and allowed to grow inside an anaerobic jar in the presence of GasPak EZ for 24 h. Cells were separated from the culture medium by centrifugation at 3650× *g* for 20 min (Centrisart^®^ D-16C, Sartorius, Göttingen, Germany), then cell pellets were washed twice in sterile water and lastly re-suspended in 240 mL of PBS pH 7.4. The suspensions were left for 2 h at room temperature on an orbital shaker (Certomat^®^ Sartorius AG, Göttingen, Germany) at 100 rpm to enable the release of the cell bound BS. The supernatant containing the BS was isolated by two sequential centrifugations at 2543× *g* for 20 min (ALC 4222MKII centrifuge, ALC International s.r.l., Milan, Italy) and any remaining cellular components were removed by filtration through a 0.22 μm pore size filter (Cellulose acetate syringe filter, Sanford, FL, USA). Removal of PBS salts and impurities was obtained through dialysis against demineralized water in a standard RC tubing (molecular weight cut-off 6000–8000 Da; Spectra/Por 1 dialysis membrane Spectrum Laboratories Inc., Rancho Dominguez, CA, USA) for 24 h at room temperature, and lastly the dialysed supernatant was freeze dried at 0.01 atm and −45 °C (Christ Freeze Dryer ALPHA 1–2, Milan, Italy).

### 2.3. Surface Activity and Critical Micelle Concentration of Surfactants

The surface-active properties of surfactants and their critical micelle concentration (CMC) were determined through the ring method using a tensiometer (K8600E Krüss GmbH, Hamburg, Germany). The surface tension (dyne/cm) was measured at room temperature as the force required to detach the platinum ring (1.9 cm diameter) from 2 mL of PBS pH 5.5 solution containing different concentrations of BC9-BS (0.0625–8.005 mg/mL), CAPB (0.005–5.275 mg/mL) and TPGS (0.02–2 mg/mL). The concentration at which surfactants change their organization from single molecules to micelles was determined by plotting the surface tension as function of the logarithm of surfactants concentration. Precisely, the CMC coincides with the intersection between the curve that describes the linear decrease in surface tension and the one that includes the points for which the increase in surfactants concentration corresponds to constant values of surface tension.

### 2.4. Chromatographic Conditions

HPLC analytical assay was performed using a Shimadzu (Milan, Italy) LC-10ATVP chromatographic pump and a Shimadzu SPD-10AVP UV–vis detector set at 244 nm. Separation was obtained on a Phenomenex (Torrance, CA, USA) Sinergy^TM^ 4 µm Hydro-RP 80Å LC column (150 × 4.60 mm) coupled to a Phenomenex Security Guard C18 guard cartridge (4 × 3.0 mm i.d., 5 μm). The mobile phase consisted of a mixture of acetonitrile/PBS pH 7.4 40:60 (*v*/*v*) and it was flushed at a rate of 0.5 mL/min. Manual injections were made using a Rheodyne 7125 injector with a 20 μL sample loop and data analysis was carried out through the CromatoPlus software (Shimadzu Italia, Milan, Italy). Because of the different purposes of this work, more than one calibration curve was obtained. The calibration curve of HC in ethanol/PBS pH 5.5 (1:1 *v*/*v*) was characterized by a drug concentration range of 2.625–105 μg/mL, a linearity coefficient (R^2^) equal to 1, and it was employed to determine HC solubility. The calibration curve of HC in PBS pH 7.4/ ethanol (80:20 *v*/*v*), obtained with drug concentrations ranging from 0.10 μg/mL to 41.52 μg/mL, showed a good linearity (R^2^ = 1) and it was used to evaluate the drug permeated during the in vitro diffusion studies. Limits of detection (LOD) and quantification (LOQ) were 0.14 μg/mL and 0.41 μg/mL, respectively.

### 2.5. Surfactants Solubilizing Activity

To investigate surfactants ability to increase solubility of HC, an excess amount of drug was dispersed in PBS pH 5.5 in absence (CTRL sample) or in presence of BC9-BS, CAPB or TPGS at two different concentrations: below and above their CMC, which corresponded to half and five-fold the CMC, respectively. Dispersions were left under stirring for 48 h at room temperature (25 °C), and subsequently were subjected to centrifugation at 5890× *g* for 15 min (Microspin 12, Biosan, Riga, Latvia) and filtration through syringe filters 0.22 µm cut-off to remove the fraction of undissolved drug. The samples obtained through this procedure were used as such for the in vitro permeation study (see Section 2.7), whereas, to assess the maximum solubility of HC in the presence of surfactants, specimens were diluted 1:1 (*v*/*v*) in ethanol prior to HPLC analytical assay.

### 2.6. Surfactants Interaction with Mucin

The ability of surfactants to interact with the main component of the mucus gel layer was investigated through turbidimetric measurement of a suspension containing mucin and surfactants at concentrations below and above the CMC, as reported by Abruzzo et al., 2018 [14] with some modifications. Mucin previously dialyzed and lyophilized, as reported in Section 2.7, was used to prepare a mucin dispersion (0.08 % *w*/*v*) in PBS pH 5.5. After stirring for 6 h, mucin dispersion was centrifuged (3310× *g*, 20 min) to remove the excess amount of mucin. The mucin obtained was thus mixed at a 1:4 volume ratio with the suspension containing surfactants in PBS pH 5.5, and then vortexed for 1 min. The turbidity of the samples was measured at 650 nm through an UV–visible spectrophotometer (UV-1601 Shimadzu, Milan, Italy). The absorbance (*ABS*) of mucin dispersion itself and surfactants suspensions without mucin were measured as references. In fact, data were reported as the percentage increase in sample absorbance in the presence of mucin with respect to the same sample without mucin, according to the following equation:

(1) $$\%ABS=\frac{ABS\;sample\;with\;mucin-ABS\;mucin}{ABS\;sample\;without\;mucin}*100\;$$

### 2.7. Surfactants Permeation Enhancing Properties

To evaluate surfactants ability to act as permeation enhancers, diffusion studies of HC in the presence of BC9-BS, CAPB or TPGS at concentrations below and above the respective CMC (half and five-fold the CMC, respectively) were performed using Franz-type static glass vertical diffusion cells (15 mm jacketed cell with a flat-ground joint and clear glass with a 12 mL receptor volume; diffusion surface area = 1.77 cm^2^) equipped with a V6A Stirrer (PermeGearInc., Hellertown, PA, USA). Diffusion studies were conducted across different in vitro barriers:

1. PermeaPad^®^ barrier (InnoMe GmbH, Espelkamp, Germany), which had already been used to predict the nasal absorption of drugs [21], and thus was here employed to simulate the nasal epithelium;
2. PermeaPad^®^ barrier functionalized with the addition of an artificial mucus layer on its surface, that enabled us to better mimic the in vivo conditions of the nasal cavity;
3. Sheep nasal mucosa, that was exploited due to its similarity to the human one in terms of morphology [22] and because it was found adequate in previous studies [23,24].

The different membranes, PermeaPad^®^ barrier, PermeaPad^®^ barrier functionalized with a mucin layer and sheep nasal mucosa, were clamped between the receptor and donor compartments. The receptor medium was composed of 12 mL of the mixture PBS pH 7.4/ethanol (80:20 *v*/*v*), previously sonicated to avoid air bubble formation beneath the membranes, thermostated at 35 ± 1 °C thanks to a surrounding jacket and maintained under constant stirring to ensure a uniform distribution of the diffused drug. The temperature was chosen in accordance with previous in vitro diffusion studies aimed to investigate nasal drug delivery [21]. Then, 300 µL of the control sample and the samples containing surfactants at the two different concentrations tested, which were obtained as described in Section 2.5, were added to the donor compartment.

Diffusion studies were performed over 5 h, during which samplings were made pipetting 200 µL of the acceptor phase (replaced with fresh acceptor medium) every 15 min for the first 2 h and every 30 min for the subsequent 3 h. The permeability coefficient (*K*p) was calculated according to the following equation [7]:

(2) $$Kp=\frac{dM}{dt}\times \frac{1}{S\times C0}$$

where *dM/dt* (μg/s) is the slope at the steady state period, *S* (cm^2^) is the diffusion surface area and *C*0 (μg/mL) is the initial drug concentration within different samples. To better explain the influence of the different surfactants on HC permeability across the in vitro models, the enhancement ratio (*ER*) was calculated on the basis of the following equation [25]:

(3) $$ER=\frac{Kp\;with\;surfactants}{Kp\;control\;sample}$$

For the preparation of the functionalized PermeaPad^®^ barrier, mucin was dispersed in ultrapure water at the concentration of 50 mg/mL and subjected to dialysis overnight using standard RC tubing (molecular weight cut-off 6000–8000 Da), to remove those mucus glycoproteins characterized by a low molecular weight. These, in fact, could potentially diffuse across the PermeaPad^®^ barrier, hindering the possibility to produce a stable mucus gel layer over the time. The purified mucin dispersion was freeze dried and the powder obtained was stored at +2–8 °C until use. When diffusion studies were performed, 200 µL of purified mucin dispersion in PBS pH 5.5 (50 mg/mL) were placed on the top of the PermeaPad^®^ barrier and left to equilibrate for 5 min prior to sample addition in the donor compartment.

The nasal mucosa was obtained from a local slaughterhouse and the tissue used for the permeation studies was precisely the one excised from the nasal turbinates. First, nasal turbinates were separated from the septum using forceps and scalpel, then the mucosa was carefully detached from the adhering cartilaginous tissue and abundantly washed with NaCl 0.9% (*w*/*v*). The excised tissue was placed on a nitrate cellulose filter characterized by 0.45 µm pore size (Sartorius, Göttingen, Germany), with the epithelium side in direct contact with the filter itself and the mucosal side facing upwards, and finally stored in aluminium foils at −20 °C until use.

### 2.8. Statistical Analysis

All results are shown as mean ± standard deviation (SD) and SD was calculated from the values of three independent experiments. Data from all experiments were analyzed using a *t*-test, and differences were deemed significant for *p* < 0.05.

## 3. Results and Discussion

### 3.1. BC9-BS as Surface-Active Agent and Critical Micelle Concentration

**Figure 1:**
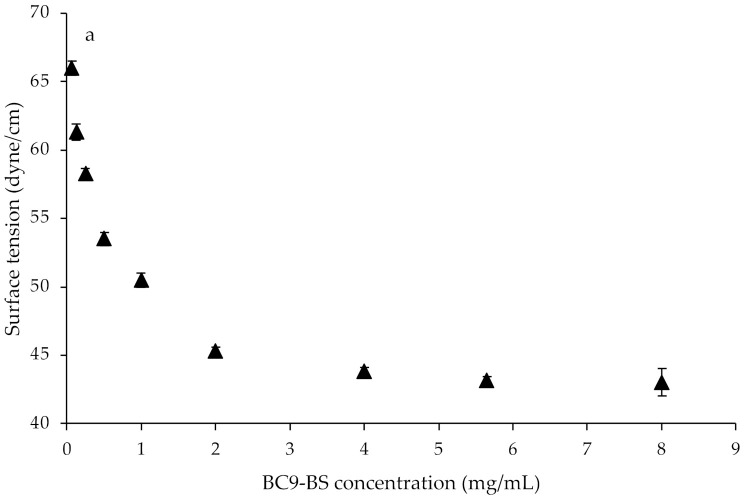

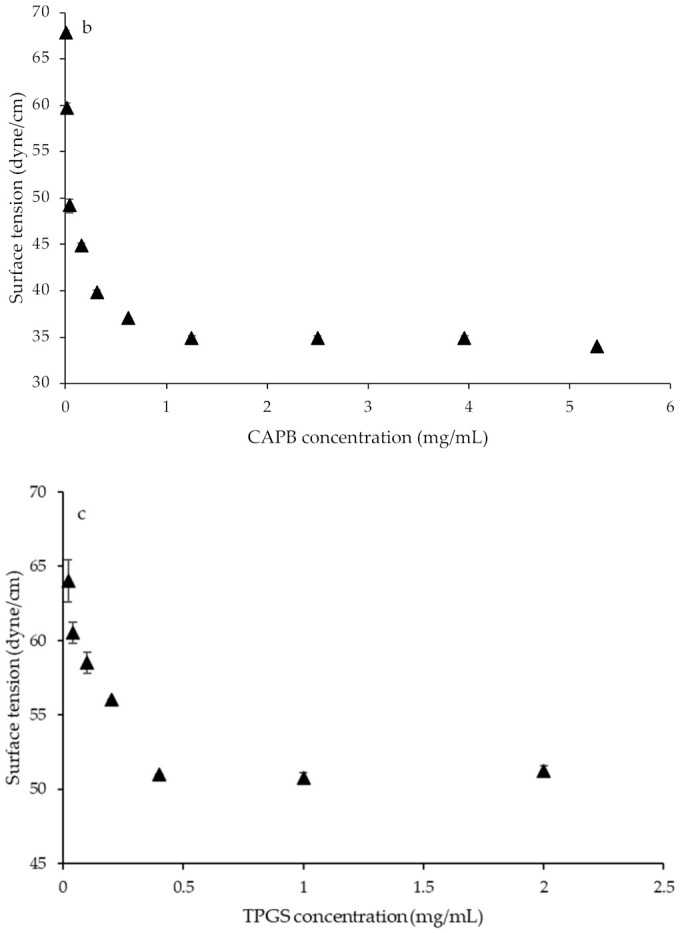
Surface tension values as function of (**a**) biosurfactant from *L. gasseri* BC9 (BC9-BS), (**b**) Cocamidopropyl betaine (CAPB) and (**c**) D-α-tocopheryl polyethylene glycol 1000 succinate (TPGS) concentrations (mg/mL). Data are plotted as mean values of surface tension (dyne/cm) ± SD (*n* = 3).

Beyond being natural compounds, biosurfactants (BS) are amphiphilic molecules, thus their potential applications rely on the ability to reduce surface and interfacial tension [10,12,25]. In the present study, surface-activity of BC9-BS, CAPB and TPGS was evaluated at room temperature (25 °C) and at pH 5.5 by means of a phosphate buffer used to mimic the nasal pH, which ranges from 5.5 to 6.5 in adults and from 5.0 to 6.7 in children [26]. Figure 1, which shows the surface tension plotted as a function of surfactant concentration, confirmed that all tested compounds exhibit surface-active properties. Moreover, for each of them it was possible to calculate the CMC, known as the concentration that enables the lowest stable surface tension to be reached, and after which surfactants self-assemble in micelles [9,10].

When BC9-BS was solubilized at concentrations from 0.0625 mg/mL to 8.005 mg/mL, a decrease in surface tension from 66 ± 0.5 to 43 ± 1 dyne/cm was observed and the CMC was found to be around 2 mg/mL. Data obtained are in agreement with our previously published results: the presence of electrolytes allows for a greater reduction in surface tension with respect to water, without affecting the CMC value [18]. Regarding CAPB, it was demonstrated to reduce surface tension from 67.8 ± 0.3 to 34 ± 0 dyne/cm in the concentration range of 0.005–5.275 mg/mL and it exhibited a CMC value of 1.01 mg/mL, which is in line with what has been already reported in literature [27]. Lastly, the synthetic surfactant TPGS allowed for a decrease in surface tension from 64 ± 1.4 to 51.25 ± 0.3 dyne/cm when solubilized at concentrations from 0.02 mg/mL to 2 mg/mL and it was characterized by a CMC value of 0.54 mg/mL. The latter value was found to be higher than that obtained in previous studies (0.2 mg/mL [28]), probably because of the low pH at which the measurement was conducted. In fact, it has been demonstrated that the pH can affect the micelle properties of a non-ionic surfactant, the CMC included; at a constant temperature, an increase in the CMC can be observed while decreasing the pH [29].

Based on the calculated CMC values, surfactants activities as solubilizing and permeation enhancing agents were evaluated at concentrations of half and five-fold the respective CMC. The selected concentrations were thought to be suitable to investigate the different behavior of surface-active molecules as a function of their different organization in the buffer medium: single molecules or micelles. In fact, it is known from the literature that, differently from when employed at concentrations below the CMC, surfactants used at concentrations higher than the CMC are mainly responsible for drug solubility increase rather than for drug permeability improvement [24,30]. Regarding the issue of toxicity, it is worth noting that none of the investigated surfactants was previously reported to be toxic at concentrations equal to those tested in the present study. As a matter of fact, cell viability assays using BC9-BS up to 10 mg/mL on human and murine fibroblasts indicated that BS is not cytotoxic [17]; similarly, CAPB exerted no toxic effect to NIH 3T3 cells when employed at concentrations up to 35.8 mg/mL [31] and its extensive use in detergent and cosmetic industries is well established because of its low irritative potential on the skin and mucous membranes [20]. Lastly, TPGS was exploited to develop curcumin loaded polymeric micelles intended for nose-to-brain delivery and, after treatment, neither epithelial changes nor sign of remarkable destructive effect were observed on the nasal mucosa [32].

### 3.2. BC9-BS as Solubilizing Agent

Drug solubility represents a key factor in pharmaceutical research and development, and it is even more relevant when it regards nasal drug delivery. In fact, due to the small volume of formulation that can be delivered to the nasal cavity, the administration of low water-soluble active ingredients in quantities that are sufficient to exert a therapeutic effect can be difficult [5,33]. As a result, in the present study, solubility experiments were performed to investigate whether BC9-BS and the two other surfactants used as models, CAPB and TPGS, could be employed as excipients to improve HC solubility and thus facing the limit posed by the volume restriction. Moreover, since according to the Fick’s first law the diffusion of active molecules is directly proportional to the solubility of the drug, solubility measurements were strictly necessary to evaluate HC permeation during in vitro studies [24].

**Figure 2:**
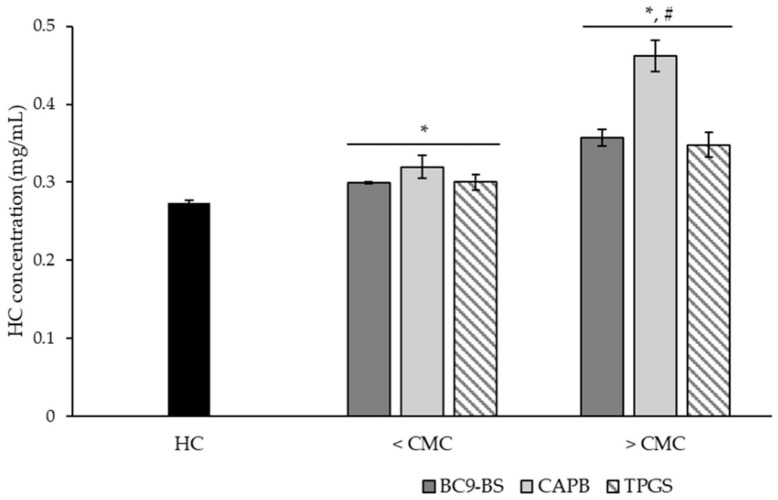
Surfactants influence on hydrocortisone (HC) solubility at room temperature (25 °C) when used at concentrations below (<) and above (>) the respective critical micelle concentration (CMC). Data are expressed as means ± SD, *n* = 3. Significance indicated by * = *p* < 0.05 with respect to HC solubility without surfactants (black bar), by # = *p* < 0.05 compared to HC solubility in the presence of the same surfactant at a concentration < CMC.

Figure 2 displays results obtained by solubilizing HC at room temperature in PBS pH 5.5 in the absence and presence of surfactants at concentrations half-fold (<CMC) and five-fold (>CMC) the respective CMC.

The maximum solubility reached by HC in the buffer solution without the addition of any of the surfactants was 0.274 ± 0.004 mg/mL, which was lower than that observed in water (0.295 ± 0.003 mg/mL [30]). This result is reasonable if taking into consideration the “salting-out” effect, which consists of the ability of inorganic salts, such as NaCl, to decrease the solubility of nonelectrolytes by increasing the polarity of water [34].

Results clearly demonstrated that all tested surfactants, when added at a concentration below their CMC, already determined a significant increase in drug solubility (*p* < 0.05) with respect to the sole HC. Such an increment was even more noticeable when surfactants were used at a concentration five-fold the CMC, reasonably because of amphiphilic molecules tendency to self-assemble in micelles, which are characterized by a hydrophobic region available for the solubilization of HC. BC9-BS, in particular, led to an increase in HC solubility up to 0.300 ± 0.001 mg/mL and 0.357 ± 0.011 mg/mL below and above its CMC respectively, confirming our previous findings: biosurfactants act as solubilizing agents and are able to interact with the drug both as single molecules (i.e., true supersaturation) and micelles [18,24].

### 3.3. BC9-BS Interaction with Mucin

**Figure 3:**
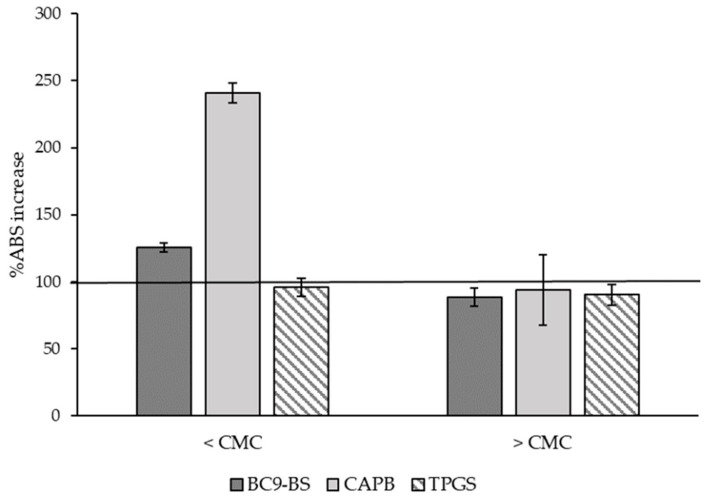
Surfactants interaction with mucin as function of their concentration. Data are reported as percentage increase of the sample absorbance (*ABS)* in the presence of mucin with respect to the same sample without mucin, and are expressed as means ± SD, *n* = 3.

Another limiting factor in nasal delivery is the mucus turnover due to the mucociliary clearance in the upper respiratory tract, that negatively influences the efficacy of liquid nasal formulations. Therefore, a main goal would be to develop innovative formulations able to increase drug residence time and adhesion to the site of administration [35]. Because of this, surfactant capability to interact with the main component of the mucus layer was investigated solubilizing the surface-active agents in PBS pH 5.5 at concentrations below and above their respective CMC, both in the presence and absence of mucin. Results reported in Figure 3 demonstrated that, below the CMC, only BC9-BS and CAPB were able to interact with mucin. The percentage *ABS* (%*ABS*) increase might be due to the electrostatic interactions between mucin glycoproteins, in particular the sialic acid residues which are deprotonated at pH values above 2.6 [35], and the positive charges contained in the investigated molecules. Neither BC9-BS nor CAPB are cationic surfactants; nevertheless, the peptidic portion of the BS contains some residues of His [14], a basic amino acid, and the betaine is characterized by a balance between positive and negative charges, because at pH 5.5 it is present in its zwitterionic form [36]. This hypothesis is in agreement with what was obtained in the case of TPGS: because of its non-ionic nature, it did not allow for a %*ABS* increase. Interestingly, when surfactants were used at a concentration five-fold their CMC, none of them were able to interact with mucin. This was not surprising for TPGS, but the fact that BC9-BS and CAPB exhibited a mucoadhesive potential exclusively as single molecules suggested that the micellar organization could hide some functional groups that were previously available for mucin interaction.

### 3.4. BC9-BS as Nasal Permeation Enhancer

Given the multiple barriers that active ingredients must overcome before reaching their target, drug absorption represents a great issue for nasal delivery. Therefore, formulation scientists frequently exploit permeation enhancers, which can increase drug absorption through the mucosal tissue by temporarily altering the nasal membrane [37]. The possibility to employ BC9-BS as an innovative absorption enhancer in the field of nasal drug delivery was investigated performing diffusion studies across different in vitro models, such as the innovative PermeaPad^®^ barrier. The latter belongs to the cell-free permeation tools class and, since it is made of phospholipids sandwiched between two support sheets, mimics the cell membrane and thus it is referred as a biomimetic membrane [38]. PermeaPad^®^ is considered a new cost-effective, easy to use and reliable system for drug permeability screening characterized by a good shelf-life and resistance to pH variations [39]. Moreover, its ability to predict passive drug permeability in the presence of surfactants, co-solvents and simulated fluids makes it suitable to study enabling formulations [40]. Initially, the PermeaPad^®^ barrier was developed to investigate the permeability of drugs intended for oral delivery; then, it was further evaluated for its ability to foresee buccal [41] and nasal permeability [21].

**Figure 4:**
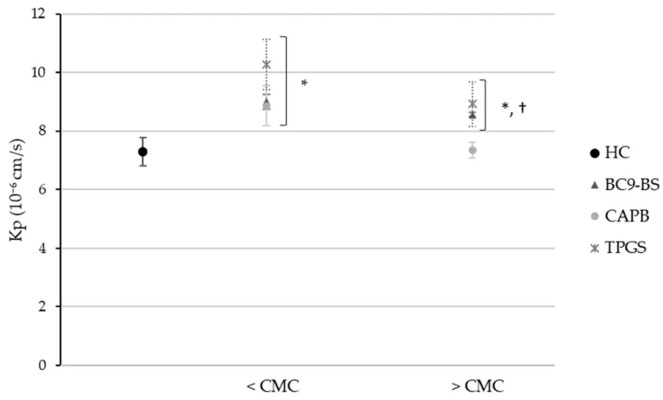
HC permeability across the PermeaPad^®^ barrier in the absence and presence of surfactants at concentrations below (<) and above (>) their CMC. Data are expressed as means ± SD, *n* = 3. Significance indicated by * = *p* < 0.05 with respect to Kp of HC without surfactants, and by † = *p* < 0.05 compared to Kp of HC in presence of CAPB > CMC.

Figure 4 shows HC apparent permeability coefficient (Kp) across the PermeaPad^®^ barrier in the absence and presence of surfactants used at concentrations below and above their CMC, half-fold and five-fold respectively. When used at a concentration below the CMC, all tested surfactants were able to significantly (*p* < 0.05) increase drug permeability with respect to the control. In particular, BC9-BS improved HC permeability from 7.300 ± 0.480 10^−6^ cm/s in the control sample to 9.000 ± 0.257 10^−6^ cm/s, thus demonstrating the biosurfactant ability to act as a permeation enhancer. Considering the samples containing surfactants at a concentration five-fold their CMC, the absorption enhancing effect was still detectable only for BC9-BS and TPGS. Conversely, HC permeability in the presence of betaine was found to be significantly lower with respect to that of both the BS and the non-ionic surfactant, and it was also comparable to that of the control (*p* > 0.05). A decrease in drug permeability in the presence of a surfactant used at a concentration above the CMC with respect to that obtained with the same surface-active molecule but at a concentration below the CMC is not something new. Indeed, Abruzzo et al. [30] had already observed this phenomenon while studying the ability of some surfactants produced from itaconic acid to improve HC permeation across the skin. In the case of the present study, the different CAPB behavior compared to the other two surfactants might be the result of a higher HC entrapment into micelles. Probably, since micelles act as a drug reservoir, they tend to gradually release HC in the buffer medium, thus slowing down its diffusion across the membrane.

**Figure 5:**
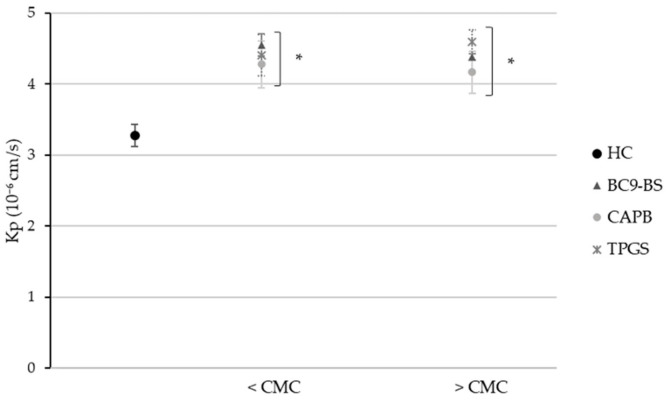
HC permeability across the PermeaPad^®^ barrier functionalized with a mucin layer in the absence and presence of surfactants at concentrations below (<) and above (>) their CMC. Data are expressed as means ± SD, *n* = 3. Significance indicated by * = *p* < 0.05 with respect to Kp of HC without surfactants.

The PermeaPad^®^ barrier only reproduces the epithelium of the mucosal tissue, thus it does not take into account the mucus gel layer that covers the nasal cavity. To deepen the ability of BC9-BS to improve nasal absorption of HC, the PermeaPad^®^ model was enriched with an artificial mucin layer. The latter was obtained through mucin dialysis and subsequent freeze-drying to remove from the initial mixture of glycoproteins, those low molecular weight molecules able to diffuse across the PermeaPad^®^, thus preventing the possibility to maintain a stable mucus layer over the time. The results collected from the in vitro permeation study across the PermeaPad^®^ and mucin system highlighted the impact of the mucus layer on the diffusion of the model drug (Figure 5). As a matter of fact, HC permeability decreased from 7.300 ± 0.480 10^−6^ cm/s in the absence of mucin, to 3.275 ± 0.153 10^−6^ cm/s in the presence of the mucus layer, consequently underlining the negative influence of mucus on drug absorption. This phenomenon had already been observed by Falavigna et al. [7], who developed a mucus-covered artificial permeation membrane by pipetting a mucin dispersion on the top of a PVPA (phospholipid vesicles-based permeation assay) barrier, clarifying the impact of the mucus layer on the absorption of hydrophilic and lipophilic drugs. Moreover, our result was in line with previously reported data, which elucidated the tendency of lipophilic molecules to non-specifically bind to hydrophobic regions in mucin glycoproteins, resulting in drug diffusion hindrance through the mucus layer [35]. Despite the additional obstacle towards HC permeation, BC9-BS significantly (*p* < 0.05) increased drug permeability with respect to the control at both the concentrations tested, reaching values of 4.550 ± 0.159 10^−6^ cm/s and 4.383 ± 0.047 10^−6^ cm/s at half and five-fold its CMC, respectively. Moreover, the biosurfactant was demonstrated to be as effective as CAPB and TPGS in improving drug absorption. Surprisingly, CAPB, that did not appear as a permeation enhancing molecule on the PermeaPad^®^, in the presence of the mucin layer allowed for a greater increase in HC permeability with respect to the control.

For a better understanding of the permeation enhancing properties of the tested surfactants when the mucus layer was included in the barrier system, the enhancement ratio (*ER*) was evaluated. Since on both PermeaPad^®^-based models the greater absorption enhancing effect was observed when surfactants were used at the lowest concentration tested, the *ER* value was calculated for each compound only when used at a concentration below the CMC. Table 1 shows that only BC9-BS and CAPB exhibited a higher *ER* value across the PermeaPad^®^ barrier functionalized with mucin compared to the simple PermeaPad^®^. It is known that the mucus layer acts as a barrier towards the diffusion of foreign entities, such as drugs and particles, exploiting two main mechanisms: interaction and size filtering [8]. The first includes those weak interactions that occur between the mucus and the investigated molecule, whereas size filtering depends on the mucus mesh cut-off, which can avoid the diffusion of large entities [7]. Thus, probably, BC9-BS and CAPB, in virtue of their interaction with mucin (see Section 3.3), can perturb the mucus layer, favoring HC diffusion toward the PermeaPad^®^ membrane.

**Figure 6:**
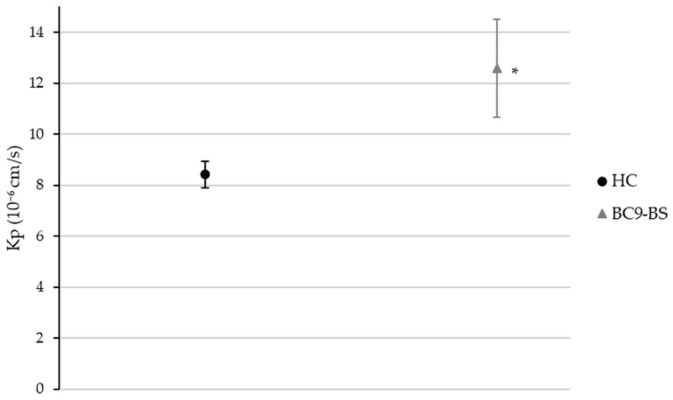
HC permeability across the sheep nasal mucosa in the absence and presence of BC9-BS at a concentration below the CMC. Data are expressed as means ± SD, *n* = 3. Significance indicated by * = *p* < 0.05 with respect to the control.

Since BC9-BS was the surfactant characterized by the higher *ER* value on the PermeaPad^®^ and mucin model, its absorption enhancing properties were also investigated performing diffusion studies across the sheep nasal mucosa. Furthermore, since no significant differences (*p* > 0.05) in drug permeability were observed using the biosurfactant at a concentration below or above its CMC, neither with the PermeaPad^®^ model nor with the PermeaPad^®^ and mucin one, the experiment was conducted using BC9-BS only at a concentration of half its CMC. Figure 6 shows that BC9-BS was able to improve HC permeability across the sheep nasal mucosa too, as it was predicted by previous in vitro models. Despite the high standard deviations, that are a consequence of the inter-animal and inter-membrane differences, the Kp coefficient of HC in the presence of the biosurfactant was confirmed to be significantly improved with respect to the control (*p* < 0.05).

## 4. Conclusions

The use of biosurfactants (BS) for drug delivery purposes has been attracting great interest in recent years and, as a result, proofs of their applicability as enhancer molecules for nasal administration of drugs have already been published [42,43]. Nevertheless, it can be stated that this is the first study that evaluated the employment of biosurfactants isolated from a human *Lactobacillus* strain as a natural excipient and absorption enhancer for nasal drug delivery. BC9-BS was able to increase HC solubility, as well as drug permeability, across both PermeaPad^®^-based models and, at a concentration below its CMC, it allowed for a greater HC diffusion across the excised animal tissue too. Of note, BC9-BS activity as a solubilizing agent and absorption enhancer was already visible at the lowest concentration tested, the same at which the biosurfactant was also found to be able to interact with the main component of the nasal mucosa, i.e., mucin.

With respect to the surfactants used as reference, the BS shows some features that are common to both: the biosurfactant shows a mucoadhesive potential as the betaine does and it exhibits a solubilizing activity that is equal to that of the non-ionic surfactant. However, considering the permeation across the PermeaPad^®^ barrier functionalized with a mucin layer, which is the in vitro model that better mimics the in vivo conditions of the nasal cavity, BC9-BS proves to be the surface-active molecule with the higher permeation enhancing effect. Along with its demonstrated effectiveness, BC9-BS is considered more advantageous than the synthetic equivalents in virtue of its natural origin, because it is produced from renewable sources and, since it is a biosurfactant, it is reported to be more easily biodegraded and less toxic than chemical surfactants.

For a better comprehension of biosurfactants role as innovative excipients, future studies should investigate whether BS are also effective at improving the delivery of drugs with different physico-chemical features. Moreover, it could be interesting to evaluate the effect of BS inclusion in different kind of formulations aimed to improve nasal administration of active ingredients.

## Figures and Tables

**Table 1:** Enhancement ratio (*ER*) of surfactants employed at a concentration below the CMC using the PermeaPad^®^ or PermeaPad^®^ functionalized with a mucin layer.

Sample	PermeaPad^®^	PermeaPad^®^ and Mucin
BC9-BS	1.23	1.39
CAPB	1.21	1.31
TPGS	1.41	1.35

## Data Availability

Data is contained within the article.
